# A Novel Six Consecutive Monthly Doses of Palivizumab Prophylaxis Protocol for the Prevention of Respiratory Syncytial Virus Infection in High-Risk Preterm Infants in Taiwan

**DOI:** 10.1371/journal.pone.0100981

**Published:** 2014-06-27

**Authors:** Hsin Chi, Chyong-Hsin Hsu, Jui-Hsing Chang, Nan-Chang Chiu, Han-Yang Hung, Hsin-An Kao, Li-Chuan Weng, Fu-Yuan Huang, Yu-Ying Chiu, Luan-Yin Chang, Li-Min Huang

**Affiliations:** 1 Department of Pediatrics, Mackay Memorial Hospital, Taipei, Taiwan; 2 Graduate Institute of Clinical Medicine, National Taiwan University College of Medicine, Taipei, Taiwan; 3 Department of Nursing, Mackay Junior College of Medicine, Nursing, and Management, Taipei, Taiwan; 4 Department of Medicine, Mackay Medical College, New Taipei City, Taiwan; 5 Department of Medical Research, Mackay Memorial Hospital, Tamshui, Taiwan; 6 Department of Clinical Virology of Laboratory Medicine, Mackay Memorial Hospital, Taipei, Taiwan; 7 Department of Pediatrics, National Taiwan University Hospital, College of Medicine, National Taiwan University, Taipei, Taiwan; University of Iowa, United States of America

## Abstract

**Background:**

Respiratory syncytial virus (RSV) circulates year round in Taiwan. A novel six consecutive monthly doses of palivizumab for RSV prevention protocol has been approved for high risk preterm infants since December 2010. This study aimed to determine the clinical effectiveness and safety of this novel protocol for the prevention of RSV infection.

**Methods:**

From April 2011 to March 2013, we enrolled infants born at ≤28 weeks gestation and infants born at ≤35 weeks gestation with chronic lung disease (CLD) who received palivizumab prophylaxis as study group and followed up for 12 months. Historic control, those who were born and followed up between July 2000 and June 2008, were retrieved for propensity score matching. Primary endpoint was RSV-related hospitalization, and secondary endpoints included the length of hospital stay and intensive care unit (ICU) care.

**Results:**

We enrolled 127 infants (108 infants born at ≤28 weeks and 19 infants born at 29–35 weeks with CLD). They completed 6-dose palivizumab as scheduled. Among the study group, the RSV-related hospitalizations were 2 (1.6%) within 6 months and 5 (3.9%) within 12 months after discharge. We matched 127 infants in the control group with 127 infants in the study group by propensity score matching. The reduction of RSV-related hospitalization rates were 86% (10.2% vs 1.6%, *p* = 0.002) within 6 months after discharge and 78% (15.7% vs 3.9%, *p* = 0.004) within 12 months after discharge. Compared to the control group, the rate of ICU care significantly decreased from 7.1% to 0.8% (*p = *0.024) within 6 months after discharge and from 7.9% to 0.8% (*p = *0.014) within 12 months after discharge. Adverse events were recorded in 6.4% injections.

**Conclusions:**

Six monthly intramuscular administration of palivizumab is effective for prevention of RSV hospitalization in regions with no single seasonal peak of RSV infection such as Taiwan.

## Introduction

Respiratory syncytial virus (RSV) is a major cause of bronchiolitis and pneumonia in infants and young children [1], [2]. The peak incidence of RSV related hospitalization is between the second and the sixth month of age [2]. In countries with temperate climates that have well defined RSV seasons, approximately 50% of RSV-related hospitalization occurs in infants younger than 6 months [3], [4]. In Taiwan, the population-based annual incidence of RSV-associated hospitalization was highest under 6 months of age and 70% of the patients with RSV-associated hospitalization are infants [5]. Predisposing conditions for the development of serious RSV disease include young age, prematurity, chronic lung disease (CLD), congenital heart disease (CHD), neuromuscular impairment, immunodeficiency, and Down syndrome [6], [7]. Besides, RSV infection causes most of rehospitalization in preterm infants [8], [9] and represents a major burden to public healthcare worldwide [10].

Palivizumab is a humanized IgG1 monoclonal antibody specific for the F-glycoprotein on the surface of RSV [11]. It was licensed in June 1998 by the US Food and Drug Administration for prevention of serious lower respiratory tract infections (LRTIs) caused by RSV among subsets of infants and young children who are at high risk for severe RSV disease [12], [13]. In the United States, the American Academy of Pediatrics (AAP) recommends giving a maximum of 3 to 5 monthly doses of palivizumab to infants, depending on risk factors, gestational age, and time of birth relative to RSV season, with the first dose in most areas administered in November and the last dose in March [14]. Although monthly administration of palivizumab for prophylaxis in high-risk infants to prevent RSV infection is proved to be effective, it needs to be timed according to local circulation patterns of the virus [15]. The substantial cost of immunoprophylaxis with palivizumab highlights the need to define the RSV season as accurately as possible to ensure that doses are given in a timely and efficient manner [16], [17]. In temperate climates, RSV infections typically occur as well-defined community outbreaks during late autumn, winter, and early spring months, making it possible to restrict the use of immunoprophylaxis to these outbreak periods [18]–[20].

Taiwan, the country is centered near 24°N latitude and 121°E longitude. We found the RSV infections occurred throughout the year and with peaks in spring and fall in northern Taiwan [21], [22] and without significant seasonality in southern Taiwan [23]. The so-called “RSV season” for RSV immunoprophylaxis in high-risk infants in Taiwan might be different from other country with seasonal peaks of RSV infections such as United States [13].

Although palivizumab is approved in more than 60 countries and has been approved for use in the United States and Europe for more than a decade, it is not approved in Taiwan till 2008. According to the seasonality and the affected chronological age of RSV infection, a novel RSV prevention protocol for high-risk preterm infants has been proposed by the Taiwan Society of Neonatology and reimbursed by National Health Insurance since December 1, 2010. The protocol consists of six intramuscular injections of palivizumab given at monthly intervals to infants ≤28 weeks gestational age; and infants 29 through 35 weeks gestational age with chronic lung disease (CLD). The first dose of palivizumab is administered 3–5 days before the baby is discharged. To the best of our knowledge, this is the most extensive RSV prophylaxis program in a country without clearcut RSV seasonality.

The aim of this study is to determine the clinical effectiveness and safety of the novel six consecutive monthly doses of palivizumab prophylaxis protocol for the prevention of RSV infection in high-risk preterm infants.

## Materials and Methods

### Ethics statement

This study was conducted in accordance with the Declaration of Helsinki and complied with Good Clinical Practice according to International Conference of Harmonization guidelines. Ethical approval for this study was obtained from the Mackay Memorial Hospital Ethics Committee (10MMHIS015). Written informed consents from parent were obtained for all subjects.

### Study group

This prospective study was conducted at Department of Pediatrics of Mackay Memorial Hospital. The infants of study group were who received palivizumab prophylaxis according to protocol suggested by the Taiwan Society of Neonatology and were enrolled from April 1, 2011 to March 31, 2013. This protocol recommended six doses monthly intramuscular injections of 15 mg/kg of palivizumab to infants who were born at ≤28 weeks gestation and infants born at ≤35 weeks gestation with CLD. The first dose of palivizumab is administered 3–5 days before the baby was discharged. The CLD was defined as requiring supplemental oxygen therapy at 36 weeks postconceptional age. The primary endpoint was hospitalization due to RSV infection at any point from the first dose through 12 months after the first dose of palivizumab, which included hospitalization with respiratory problems and a positive RSV test, development of new RSV infection in hospitalized subject, or death from RSV infection. Secondary endpoints included length of hospital stay, intensive care unit (ICU) care, and respiratory supports. Nasopharyngeal aspirations were obtained from all hospitalized subjects with clinical symptoms of respiratory tract infections. RSV infection was confirmed either using direct immunofluorescence test or viral culture from nasopharyngeal aspiration. Compliance of palivizumab prophylaxis was evaluated by recording the actual intervals of doses inoculated to calculate the percentage of the dosing interval between 25–35 days. The adverse events of injections including local reactions such as erythema, swelling, local heat and pain on injection site and systemic reactions such as fever, cough, rhinorrhea, vomiting, diarrhea and irritability were actively captured and recorded in case report files.

### Subject enrollment and follow-up

The eligible preterm infants were enrolled by the physician and/or research nurse before discharge. The parents or legal guardians were given an information package and consent form for review. Once consent was obtained, the research nurse completed an enrollment form to collect baseline data, including patient demographics, prior medical history, season when discharge, neonatal course, and details of palivizumab administration. In addition to the monthly physician visit on injection day, the research nurse also contacted the parents or legal guardians monthly either in person at clinic visits or by telephone for the 12 months following the first injection. Using these methods we obtained data on palivizumab administration, changes in baseline information, and specific facts regarding possible respiratory infections after the last contact. Taiwan Central Weather Bureau defines spring is March to May, summer is June to August, autumn is September to November, and winter is December to February.

Adverse events were defined as events reported from the first dose of palivizumab through 30 days following the final injection regardless of suspected causality. Adverse events were recorded by study nurse from inquiry of family, the events were recorded as “any” once they reported by family and as “ severe” if they needed medical visit. In the event of a hospitalization, the hospital records were reviewed by the research nurse for reason of hospitalization, length of hospital and intensive care unit (ICU) stay, and respiratory support by mechanical ventilation. The medical events for all outpatient clinics visits, emergency visits, and hospitalizations for respiratory symptoms in the past month were recorded at each of the monthly follow-up contacts by study nurse.

### Historical control group

To assess the effectiveness of prevention guidelines, baseline RSV hospitalization rates before the implementation of palivizumab prophylaxis were calculated. We retrospectively reviewed the charts of the premature babies who were born and followed up at the Department of Pediatrics of Mackay Memorial Hospital between July 2000 and June 2008. RSV infection was also confirmed either by direct immunofluorescence test or viral culture. They were also categorized into in infants ≤28 weeks gestational age and infants ≤35 weeks gestational age with CLD. Their hospitalizations due to RSV infections during 12 months after discharge were recorded. The effectiveness of palivizumab was evaluated by using the reduction of hospitalization rate for RSV infection between study group and control group in 2 periods: the first 6 months and within 12 months after the first dose of palivizumab inoculation. Taiwan Central Weather Bureau defines spring is March to May, summer is June to August, autumn is September to November, and winter is December to February.

### Statistical analyses

Data were summarized using descriptive statistics. All subjects who received palivizumab were included for analyses. Continuous variables were expressed as median with interquartile range (IQR) and were compared using Student’s t test or Mann-Whitney U test. Categorical variables were presented as frequency and percentage and were compared using chi-square or Fisher’s exact test, as appropriate. We conducted the propensity score matching and regression analysis to reduce selection bias and confounding bias [24]. We included gender, gestational age, birth weight, postnatal age when first discharge, periventricular leukomalacia (PVL), CLD and CHD into our propensity score model. To evaluate time to hospitalization for RSV infection, we constructed Kaplan-Meier survival curves stratified by Palivizumab prophylaxis. To determine any factors that may affect time to RSV hospitalization, a Cox proportional hazards analysis was conducted using a backwards conditional method. Results are presented in terms of hazard ratios (HR), with 95% confidence intervals (95% CI). A *P* value of <0.05 was considered significant. Propensity score matching was performed with the contributed R package “MatchIt”, and survival analysis was conducted with the “survival” package with R version 3.0.1 (R Foundation for Statistical Computing, Vienna, Austria).

## Results

The study group included 127 infants (108 infants were ≤28 weeks gestational age and 19 infants were 29–35 weeks gestational age with CLD). They all completed their 6-doses palivizumab protocol. The historical control group consisted of 347 infants, including 284 infants were ≤28 weeks gestational age and 63 infants were 29–35 weeks gestational age with CLD. The baseline characteristics of the study group and control group are shown in Table 1. Compared to the control group, the infants of study group were female predominant (*p* = 0.01), older at discharge (*p*<0.001), lower birth weight (*p* = 0.02) and with more CLD (*p* = 0.01) and periventricular leukomalacia (PVL) (*p* = 0.003).

**Table 1:** Baseline characteristics of subjects before and after propensity score matching.

Characteristics	Before matching			After matching		
	Study group(n = 127)	Control group(n = 347)	*p* value	Study group(n = 127)	Control group(n = 127)	*p* value
Gender Male, No. (%)	59 (46.5)	208 (59.9)	0.01	59 (46.5)	60 (47.2)	0.90
Season when discharge			0.67			0.51
Spring, No. (%)	27 (21.3)	69 (19.9)	0.74	27 (21.3)	25 (19.7)	0.76
Summer, No. (%)	24 (18.9)	84 (24.2)	0.22	24 (18.9)	27 (21.3)	0.63
Autumn, No. (%)	41 (32.3)	108 (31.1)	0.81	41 (32.3)	49 (38.6)	0.29
Winter, No. (%)	35 (27.6)	86 (24.8)	0.54	35 (27.6)	26 (20.5)	0.19
Underlying diseases						
CLD, No. (%)	100 (78.1)	233 (67.1)	0.01	100 (78.7)	101 (79.5)	0.88
CHD, No. (%)	10 (7.9)	22 (6.3)	0.56	10 (7.9)	11 (8.7)	0.82
SGA, No. (%)	9 (7.1)	15 (4.3)	0.22	9 (7.1)	10 (7.9)	0.81
PVL, No. (%)	29 (22.8)	42 (12.1)	0.003	29 (22.8)	25 (19.7)	0.54
Multiple pregnancies, No. (%)	24 (18.9)	78 (22.5)	0.40	24 (18.9)	21 (16.5)	0.62
Gestational age, week, median (IQR)	27 (25–28)	27 (26–28)	0.19	27 (25–28)	27 (26–28)	0.74
GA ≤28 week, No. (%)	108 (85.0)	284 (81.8)	0.42	108 (85.0)	106 (83.5)	0.73
GA = 29–35 week, No. (%)	19 (15.0)	63 (18.2)	0.42	19 (15.0)	21 (16.5)	0.73
Age at discharge, day, median (IQR)	80 (64.5–102.5)	74 (56.5–89.0)	<0.001	80 (64.5–102.5)	78 (62.0–98.5)	0.32
Birth weight, gram, median (IQR)	952 (794–1116)	1008 (835–1199)	0.02	952 (794–1116)	970 (793–1172)	0.41
Propensity score, median (IQR)	-	-	-	0.31 (0.21–0.39)	0.30 (0.21–0.38)	0.63

Abbreviations: GA, gestational age; CLD, chronic lung disease; CHD, congenital heart disease; SGA, small for gestational age; PVL, periventricular leukomalacia; IQR, interquartile range; No., number.

### Compliance

A total of 762 doses of palivizumab were given during the study period and 635 dosing intervals were recorded. The dosing intervals between consecutive injections were 29.5±4.0 days, 28.8±1.8 days, 28.8±1.9 days, 28.8±1.8 days and 29.0±2.7 days between dose 1 and 2, 2 and 3, 3 and 4, 4 and 5 and 5 and 6, respectively. The mean dosing interval was 29.0±2.6 days, ranged from 21–60 days. The overall compliance on dosing interval was 97.8±2.5%, ranging from 93.7% to 100%.

### Incidence of RSV-related Hospitalization

**Figure 1:**
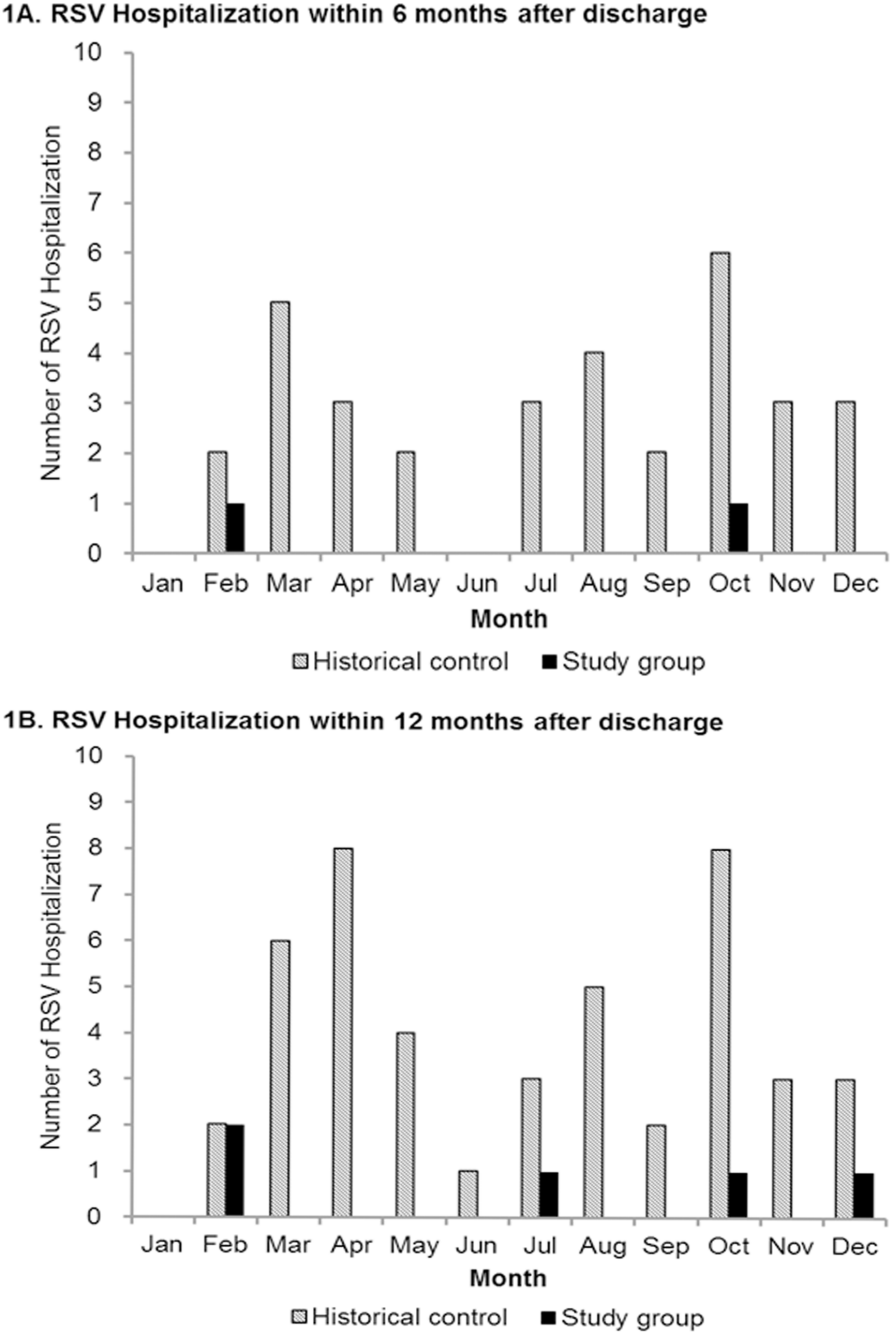
Monthly distribution of RSV-related Hospitalizations by study group and historical control group. 1A shows number of hospitalization for RSV infections within 6 months after discharge and 1B shows number of hospitalization for RSV infections within 12 months after discharge.

During the first 6 months after first palivizumab immunization, 22 subjects were hospitalized due to respiratory tract infections irrespective of the etiology and only 2 had hospitalization due to RSV infection. The timing of RSV infections of these 2 infants was 25 and 29 days after the 5th injection, respectively. The hospitalization rate for RSV infection in patients who received palivizumab was 1.6%. During the 7–12 month after first immunization, another 3 RSV-related hospitalizations occurred. Overall, a total of 5 RSV-related hospitalizations occurred within the 12 months after first immunization. The hospitalization rate for RSV infection in the palivizumab group was 3.9% during this period. In the historical control group, the overall hospitalization rate for RSV infection were 9.5% (33/347) within the first 6 months after discharge and 13.0% (45/347) within 12 months after discharge. The monthly distributions of hospitalization for RSV infections of both study group and control group are shown in Figure 1. In the study group, only one patient needed ICU care for RSV infection occurred within 6 months after discharge. Among control group, the rates of ICU care for RSV infection were 4.3% (15/347) within the first 6 months after discharge and 4.6% (16/347) within the 12 months after discharge. Compared to the control group, the rate of receiving ICU care for RSV infections was decreased from 4.3% to 0.8% (*p = *0.11) within first 6 months after discharge and was decreased from 4.6% to 0.8% (*p = *0.09) within 12 months after discharge. There was no death due to RSV infection in both study and historical control groups.

### Effectiveness of palivizumab prophylaxis

After matching, we included 127 infants in the study group and 127 infants in control. The baseline characteristics of the matched groups are shown in Table 1. There were no statistically significant differences of baseline characteristics between 2 groups and the matching balance of study and control groups improved for gender, underlying disease, and age at discharge. The reduction of RSV-related hospitalization rates were 86% (95% CI, 36%–97%) within first 6 months after discharge [13 of 127 (10.2%) vs. 2 of 127 (1.6%), *p* = 0.002] and 78% (95% CI, 40%–92%) within 12 months after discharge [20 of 127 (15.7%) vs. 5 of 127 (3.9%), *p* = 0.004]. Compared to the control group, the rate of receiving ICU care for RSV infections was decreased from 7.1% to 0.8% (*p = *0.024) within first 6 months after discharge and was decreased from 7.9% to 0.8% (*p = *0.014) within 12 months after discharge. There was no significant difference for length of hospital stay and need of the respiratory support (Table 2).

**Table 2:** The effectiveness of palivizumab prophylaxis for RSV infections within 6 months and 12 months after discharge.

Characteristics	Before matching			After matching		
	Study group(n = 127)	Control group(n = 347)	*P* value	Study group(n = 127)	Control group(n = 127)	*P* value
**Within 6 months**						
Hospitalization for RSV infection, No. (%)	2 (1.6)	33 (9.5)	0.006	2 (1.6)	15 (11.8)	0.003
Receiving ICU care, No. (%)	1 (0.8)	15 (4.3)	0.11	1 (0.8)	9 (7.1)	0.024
Intubation and IMV, No. (%)	0 (0.0)	4 (1.2)	0.52	0 (0.0)	4 (3.1)	0.13
Nasal CPAP, No. (%)	1 (0.8)	2 (0.6)	0.69	1 (0.8)	1 (0.8)	0.29
NIPPV, No. (%)	0 (0.0)	5 (1.4)	0.39	0 (0.0)	3 (2.4)	0.19
Onset after discharge, month, median (IQR)	4.5 (4.5–4.5)	1.9 (0.6–3.5)	0.44	4.5 (4.5–4.5)	1.0 (0.4–2.5)	0.076
Length of ICU stay, median (IQR)	8.0 (8.0–8.0)	10.0 (5.0–13.0)	1.0	8.0 (8.0–8.0)	10.0 (4.5–13.0)	1.0
Length of stay, day, median (IQR)	7.0 (3.5–10.5)	11.0 (7.5–17.0)	0.55	7.0 (3.5–10.5)	13 (8.0–21.0)	0.31
**Within 12 months**						
Hospitalization for RSV infection, No. (%)	5 (3.9)	45 (13.0)	0.004	5 (3.9)	20 (15.7)	0.003
Receiving ICU care, No. (%)	1 (0.8)	16 (4.6)	0.09	1 (0.8)	10 (7.9)	0.014
Intubation and IMV, No. (%)	0 (0.0)	4 (1.2)	0.52	0 (0.0)	4 (3.1)	0.13
Nasal CPAP, No. (%)	1 (0.8)	2 (0.6)	0.69	1 (0.8)	1 (0.8)	0.29
NIPPV, No. (%)	0 (0.0)	5 (1.4)	0.39	0 (0.0)	3 (2.4)	0.19
Onset after discharge, month, median (IQR)	6.9 (4.5–9.0)	3.1 (1.0–6.6)	0.059	6.9 (4.5–9.0)	2.5 (0.6–9.1)	0.24
Length of ICU stay, median (IQR)	8.0 (8.0–8.0)	9.0 (4.5–12.8)	1.0	8.0 (8.0–8.0)	9.0 (4.0–13.0)	1.0
Length of stay, day, median (IQR)	7.0 (3.5–10.5)	9.0 (7.0–14.5)	0.45	7.0 (3.5–10.5)	9.5 (6.3–18.0)	0.19

Abbreviations: CPAP, continuous positive airway pressure; ICU, intensive care unit; IMV, intermittent mandatory ventilation; NIPPV, nasal intermittent positive pressure ventilation; IQR, interquartile range; No., number.

The analyses of effectiveness of palivizumab prophylaxis by subgroup are shown in Table 3. Within 6 months after discharge, palivizumab prophylaxis had a 92% (95% CI, 36%–99%) reduction in RSV-related hospitalization rate in infants ≤28 weeks gestational age (10.4% vs. 0.9%, *p* = 0.007) and 86% (95% CI, 13%–96%) in infants ≤35 weeks gestational age with CLD (9.9% vs. 2.0%, *p* = 0.039). Within 12 months after discharge, palivizumab prophylaxis had an 80% (95% CI, 38%–93%) reduction in hospitalization rate for RSV infection in infants ≤28 weeks gestational age (16.0% vs. 3.7%, *p* = 0.005) and 79% (95% CI, 36%–93%) in infants ≤35 weeks gestational age with CLD (16.8% vs. 4.0%, *p* = 0.006). When the sample size was 127 of both groups and alpha value was 0.05, the post hoc power was 83.3% and 89.0% within 6 months and 12 months after discharge, respectively.

**Table 3:** The effectiveness of palivizumab prophylaxis for RSV infections in subgroups.

Subgroup		Study group			Control group			OR	95% CI	% of reduction		*P* value
	CLD^a^	Total	No.^b^	%	Total	No.^b^	%				95% CI	
Within 6 months												
GA ≤28wks	+	81	1	1.2	80	8	10.0	0.11	0.01–0.92	89	8–99	0.038
GA ≤28wks	–	27	0	0.0	26	3	11.5	0.12	0.01–1.18	70	–18–99	0.22
GA ≤28wks		108	1	0.9	106	11	10.4	0.08	0.01–0.64	92	36–99	0.007
GA 29–35wks	+	19	1	5.3	21	2	9.5	0.53	0.04–6.34	47	–534–96	0.61
GA ≤35wks	+	100	2	2.0	101	10	9.9	0.19	0.04–0.87	86	13–96	0.039
Overall		127	2	1.6	127	13	10.2	0.14	0.03–0.64	86	36–97	0.002
Within 12 months												
GA ≤28wks	+	81	3	3.7	80	14	17.5	0.18	0.05–0.66	82	34–95	0.010
GA ≤28wks	–	27	1	3.7	26	3	11.5	0.30	0.03–3.04	70	–204–97	0.58
GA ≤28wks		108	4	3.7	106	17	16.0	0.20	0.07–0.62	80	38–93	0.005
GA 29–35wks	+	19	1	5.3	21	3	14.3	0.33	0.03–3.52	67	–252–97	0.67
GA ≤35wks	+	100	4	4.0	101	17	16.8	0.21	0.07–0.64	79	36–93	0.006
Overall		127	5	3.9	127	20	15.7	0.22	0.08–0.60	78	40–92	0.004

a Presence (+) or absence (–) of chronic lung disease (CLD).

b Number (No.) of hospitalization due to respiratory syncytial virus infection.

Abbreviations: RSV, respiratory syncytial virus; OR, odds ratio; CI, confidence interval; GA, gestational age; CLD, chronic lung disease.

**Figure 2:**
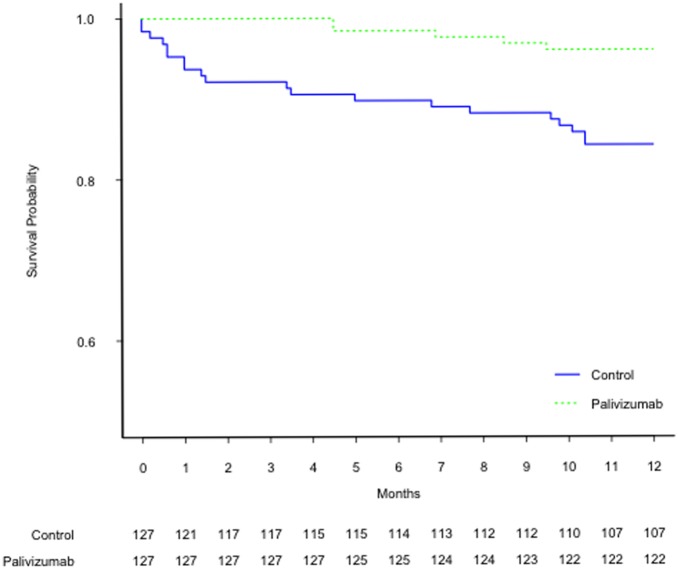
Kaplan-Meier survival curves of RSV hospitalization rate stratified by palivizumab treatment. There was statistically significant difference between palivizumab and control group (*p* = 0.001, log-rank test).

The results of multivariate Cox proportional hazards analysis indicated that palivizumab prophylaxis (HR = 0.26, 95% CI: 0.10–0.71, *p*<0.01) and age at discharge by month (HR = 1.39, 95% CI: 1.20–1.60, *p*<0.001) were significantly associated with risk for RSV-related hospitalizations. Figure 2 demonstrates the Kaplan-Meier survival curves stratified by palivizumab treatment. There was statistically significant difference between 2 groups (*p* = 0.001, log-rank test).

### Adverse events

A total of 46 out of 718 injections (6.4%) adverse events were reported during the study period; 33 events were reported from infants born at ≤28 weeks gestational age and 13 events were reported from infants born at 29 through 35 weeks gestational age with CLD (Table 4). Only 2 severe adverse events including 1 high fever and 1 irritability were reported. No adverse events led to a subject being contraindicated for the next injection of palivizumab.

**Table 4:** Adverse reactions of subjects receive palivizumab injections.

	GA ≤28wks (n = 614)				GA 29–35wks (n = 104)				Total (n = 718)			
Adverse reactions	Any		Severe		Any		Severe		Any		Severe	
	No.	%	No.	%	No.	%	No.	%	No.	%	No.	%
**Local**												
erythema	1	0.16	0	0.00	0	0.00	0	0.00	1	0.14	0	0.00
swelling	0	0.00	0	0.00	0	0.00	0	0.00	0	0.00	0	0.00
local heat	0	0.00	0	0.00	0	0.00	0	0.00	0	0.00	0	0.00
pain	1	0.16	0	0.00	0	0.00	0	0.00	1	0.14	0	0.00
**Systemic**												
fever	12	1.95	0	0.00	0	0.00	1	0.96	12	1.67	1	0.14
cough	4	0.65	0	0.00	7	6.73	0	0.00	11	1.53	0	0.00
rhinorrhea	6	0.98	0	0.00	8	7.69	0	0.00	14	1.95	0	0.00
vomiting	7	1.14	0	0.00	3	2.88	0	0.00	11	1.53	0	0.00
diarrhea	6	0.98	0	0.00	0	0.00	0	0.00	6	0.84	0	0.00
irritability	33	5.37	1	0.16	13	12.5	0	0.00	46	6.41	1	0.14

Abbreviations: GA, gestational age.

## Discussion

Our prospective study indicated that this novel 6-injection palivizumab prophylaxis guideline effectively prevents serious RSV infection in high-risk children in Taiwan. In our study, the overall 1.6% hospitalization rate for RSV infection was comparable to the 4 years US registry data which demonstrated a hospitalization rate of 1.3% in treated high-risk infants [25]. After palivizumab prophylaxis, 63–78% reduction rate of RSV hospitalizations during 12 months afterward in our study were similar to previous studies [26]–[28].

Palivizumab only confers passive immunity; therefore, protection against RSV depends on adequate palivizumab levels throughout the duration of exposure to RSV. A serum palivizumab concentration of 30 µg/mL is the proposed serologic correlate of protection; this concentration results in a 100-fold decrease in pulmonary RSV replication in cotton rat [11]. Palivizumab has been available for 15 years and is currently recommended for 3 to 5 monthly injections at 30±5 day intervals, based on pharmacokinetic evidence from RCTs [29]–[31]. The Impact-RSV study revealed that monthly intramuscular injections of palivizumab prophylaxis reduced RSV-associated hospitalizations in preterm infants and children aged ≤24 months with BPD by 55% compared with placebo (4.8% vs 10.6%, *P = *0.00004). Children with prematurity but without CLD had a 78% reduction in RSV hospitalization (8.1% vs 1.8%); children with CLD had a 39% reduction (12.8% vs 7.9%) [29].

In countries with RSV seasonality, administration of palivizumab is tailored according to time of year to maximize efficacy and minimize cost. Instead of 3 to 5 monthly doses during RSV season [14], we gave 6 injections of palivizumab for prophylaxis in the first 6 months after discharge regardless of seasonality in Taiwan. To the best of our knowledge, this is the first nation-wide RSV 6-dose prophylaxis program in a country without RSV seasonality. There are two reasons; the first is because the peak of RSV infection and the RSV-related hospitalizations in premature babies in Taiwan occurs in the first 6 months after discharge. The second reason is because that there is no so-called “RSV seasonality” in Taiwan.

The highest proportion of RSV-related hospitalization is among the 3 to 6 months of age [32]. RSV infection during the first 3 to 6 months of life has a severe course of disease when it occurs [33]. From the IRIS study [9], risk for RSV hospitalization is increased in premature infants <3 months of chronologic age at the onset of the RSV season. Tennessee Medicaid program from July 1989 to June 1993 showed that RSV hospitalizations occurred in 8.0–9.4/100 child-years in infants born prematurely in the first 6 months of life [34]. In addition to the small size of the conducting airways and incomplete development of the lung structure, the less efficient immune response may explain why age under 6 month old at the time of hospitalization is associated with a higher likelihood of being hospitalized due to RSV infection [21]. In our study, most of the babies still stayed in the hospital 3 months after birth due to prematurity. The median age of RSV-related hospitalizations in our patients was about 3 months (IQR: 1–6) after discharge, in other words, around 6 to 9 months of chronologic age.

RSV activity has been described as being continuous throughout the year in warm equatorial areas [35], [36]. In Mexico City, Hong Kong, Dhaka, Riyadh and Miami (all within 19.2–25.8 N latitude), all with a latitude similar to Taiwan, RSV activity is usually present throughout the year [37]. RSV infection has biennial pattern with peaks in spring and fall in northern Taiwan [22] but no significant seasonality in southern Taiwan [23]. Furthermore, based on the database of National Health Insurance in Taiwan, RSV related hospitalization occurs throughout the whole year with peaks in spring and fall, especially in prematurity and in patients with CHD [5]. In Taiwan, there is no exact “RSV season”.

Early termination of dosing in these individuals would place them at risk of RSV disease while the RSV season is ongoing. Non-compliance with the monthly dosing interval has been associated with increased RSV hospitalization risk [25]. Other administration problems include starting and stopping administration on time, using the correct dose, and avoiding missed doses. Monthly dosing was chosen based on the average half-life of the IgG antibody and with a goal toward reducing the proportion of patients with subtherapeutic serum concentrations. The compliance with a monthly schedule was consistently high in our study. However, palivizumab does not prevent all RSV hospitalizations; 2 children failed to be protected with scheduled prophylaxis and were hospitalized due to RSV infection.

Palivizumab injections were well tolerated. Monthly prophylaxis was not associated with significant adverse reactions. The common adverse effects of palivizumab seen in previous study included fever, runny nose, ear infection, and rash. Initial clinical trials of palivizumab reported low rates of potentially treatment-related adverse events [29]. In postmarketing surveillance studies, serious adverse events were reported to be ≤1% in large population [38], [39]. An Expanded Access Study conducted in northern hemisphere revealed that the drug-related adverse events were reported in 6.9% of children and all of the reported adverse events were mild or moderate injection site reactions [40]. Our study revealed few (6.4%) adverse events and was consistent with the favorable safety profile observed before.

During the study period, RSV infection is detected by using the direct immunofluorescence test or viral culture. They are less sensitive compared to molecular diagnostics such as real time polymerase chain reaction (RT-PCR). We speculate that even with more sensitive RT-PCR for diagnostic method, the influence to detection of RSV-related hospitalizations in both study group and historical control will be similar. This will not affect the outcome of effectiveness in this study.

The sample size of this study is not large and a placebo control group is lacking. Due to the ethical issues, we can only use historical data of RSV infections in premature babies at the same hospital as a control for the assessment of effectiveness of prophylaxis. Propensity score matching has been widely adopted when making causal inferences in non-randomized trials. The propensity score is defined as the probability of treatment assignment conditional on measured baseline covariates. In other words, treated and untreated subjects with the same true propensity score will have similar distributions of observed baseline covariates [24]. However, our study has several limitations. First, unmeasured confounding factors could not be specified in the propensity score models. Second, performance bias exists due to the lack of blinding. Such bias could lead to overestimate the treatment effect. Third, information bias is a concern for the use of historical controls in our study. The high cost of palivizumab limits its widespread use. More doses of injection in each individual will increase a financial burden. Further cost-benefit evaluation is proceeding.

In summary, palivizumab given as six monthly doses is safe and effective for prevention of RSV hospitalization in infants ≤28 weeks gestational age and in infants ≤35 weeks gestational age with CLD. Prophylaxis with this novel protocol in areas without clearcut RSV seasonality like Taiwan results in a significant reduction of RSV hospitalization with good tolerance in children at high risk.
